# The Human Toxome Project

**DOI:** 10.14573/altex.1502091

**Published:** 2015-03-04

**Authors:** Mounir Bouhifd, Melvin E. Andersen, Christina Baghdikian, Kim Boekelheide, Kevin M. Crofton, Albert J. Fornace, Andre Kleensang, Henghong Li, Carolina Livi, Alexandra Maertens, Patrick D. McMullen, Michael Rosenberg, Russell Thomas, Marguerite Vantangoli, James D. Yager, Liang Zhao, Thomas Hartung

**Affiliations:** 1 Johns Hopkins Bloomberg School of Public Health, Center for Alternatives to Animal Testing, Baltimore, MD, USA; 2 The Hamner Institute, Research Triangle Park, NC, USA; 3 ASPPH Fellow, National Center for Computational Toxicology, US EPA, Research Triangle Park, NC, USA; 4 Brown University, Pathology & Laboratory Medicine, Providence, RI, USA; 5 US EPA, National Center for Computational Toxicology, Research Triangle Park, NC, USA; 6 Georgetown University Medical Center, Washington, DC, USA; 7 Agilent Inc., Santa Clara, CA, USA; 8 Johns Hopkins Bloomberg School of Public Health, Department of Environmental Health Sciences, Baltimore, MD, USA; 9 University of Konstanz, Center for Alternatives to Animal Testing Europe, Konstanz, Germany

**Keywords:** regulatory toxicology, safety sciences, transcriptomics, metabolomics, alternative methods

## Abstract

The Human Toxome Project, funded as an NIH Transformative Research grant 2011–2016, is focused on developing the concepts and the means for deducing, validating and sharing molecular pathways of toxicity (PoT). Using the test case of estrogenic endocrine disruption, the responses of MCF-7 human breast cancer cells are being phenotyped by transcriptomics and mass-spectrometry-based metabolomics. The bioinformatics tools for PoT deduction represent a core deliverable. A number of challenges for quality and standardization of cell systems, omics technologies and bioinformatics are being addressed. In parallel, concepts for annotation, validation and sharing of PoT information, as well as their link to adverse outcomes, are being developed. A reasonably comprehensive public database of PoT, the Human Toxome Knowledge-base, could become a point of reference for toxicological research and regulatory test strategies.

## 1 Introduction

Conventional toxicity testing has relied on the exposure of laboratory animals to chemicals to examine toxic responses (apical endpoints). The testing of any single chemical has become expensive, time-consuming and exorbitant in the use of animals (Hartung and Rovida, 2009; Hartung, 2011). It is difficult for regulatory agencies to adequately examine either the numbers of new compounds entering commerce or those chemicals already in use that lack basic toxicology information (NRC, 1984; EPA, 2013a). In 2003, a US Environmental Protection Agency (EPA) report (EPA, 2003) noted that a computational toxicology program would have several advantages, primarily in prioritizing chemicals for testing and in developing predictive models for quantitative risk assessment. At the request of the EPA and the National Institutes of Environmental Health Sciences (NIEHS), the U.S. National Research Council (NRC) conducted a review of toxicity testing methods, producing the National Research Council Report *Toxicity Testing in the 21^st^ Century: A Vision and a Strategy* (TT21c) (NRC, 2007). The report has prompted a number of activities for modernizing regulatory toxicology. TT21c called for embracing new technologies and basing assessments on toxicological mechanisms. Toxicity testing using *in vitro* results from toxicity pathway assays in human cells promises to make testing faster, less costly, more humane and more relevant by focusing on human biology and exposure. TT21c proposed that a core suite of tools, including medium- and high-throughput *in vitro* screening, computational toxicology, systems biology and both toxicity pathway and pharmacokinetic modeling, would form the basis of these new test methods. Perspectives in both toxicological sciences (Andersen and Krewski, 2009) and risk analysis (Krewski et al., 2009) outlined the TT21c vision and a series of 15 responses from experts in toxicology, regulatory sciences and risk assessment provided commentaries (Andersen and Krewski, 2010; Krewski et al., 2009).

Within a year, the EPA, the National Toxicology Program (NTP) at NIEHS and the National Chemical Genomics Center (NCGC) announced a collaboration (Tox-21) to implement the key recommendations of the report (Collins et al., 2008). In 2007, EPA’s Computational Toxicology research set out to solve this problem through a multi-year effort called Toxicity Forecaster (ToxCast™). ToxCast™ uses high-throughput screening (HTS) assays to expose living cells or isolated proteins to chemicals to screen them for changes in biological activity that may suggest potential toxic effects (Kavlock et al., 2012; Judson et al., 2010). By 2013, ToxCast™ evaluated over 2,000 chemicals from a broad range of sources in more than 700 HTS assays and approximately 300 signaling pathways (EPA, 2013b). As part of the collaboration with the Human Toxome Project, ToxCast™ is evaluating the connection between perturbations observed in the HTS assays and potential adverse responses.

Simultaneously, EPA has been working on exposure prediction models (ExpoCast) for thousands of chemicals based on manufacture and use information (Wambaugh et al., 2013). Together, these can be used for risk-based chemical prioritization, e.g., for EPA’s endocrine disruption screening program (EPA, 2013a). ToxCast™ partners include other government agencies, industry, academia and NGOs (EPA, 2013a). The iCSS Dashboard was launched in 2013 to facilitate public access to the ToxCast™ data (EPA, 2013a). These programs are remarkable for the breadth of their assessments, quality assurance and transparency, including public involvement and data sharing. However, they are necessarily based on existing knowledge of relevant mechanisms.

Work at The Hamner Institute has utilized a number of case studies to identify the steps needed for immediate, wholesale changes to current practices (Adeleye et al., 2014). First, the focus has been on developing the specific safety assessment tools for interpreting *in vitro* test results and then using this *in vitro* toxicity information directly for setting regulatory standards. Second, the emphasis is on learning by doing. Many key issues relevant to the use of *in vitro* toxicity pathway assays for safety assessment will become apparent after completing the first two or three case studies. Most of the issues will become clear and expansion to other pathways will move along more quickly.

Some challenges become clear by simply looking at the anticipated risk assessment applications. With cell-based test methods, there are no specific apical responses on which to conduct a “traditional” risk assessment (Andersen and Krewski, 2010). The process, based on *in vitro* assays, estimates regions of exposures that should not cause excessive pathway perturbations in exposed populations. The definition of excessive perturbation will require *in vitro* assays that provide read-out at differing levels of severity and the ability to differentiate compensatory from adverse responses. Dose-response assessment from these assay results require integration of multiple data streams to infer the structure of the signaling circuitry and its dynamic response with increasing levels of perturbation. Dynamic, dose-response characteristics of toxicity pathways provide the grist for completing computational systems biology pathway models to assess shapes of dose response curves at low exposures (Zhang et al., 2013, 2010). Some initial toxicity pathway case studies include estrogen signaling in uterine cells^1^, p53-mediated DNA damage responses in HT-1080 cells, a human fibrosarcoma cell (Sun et al., 2013; Zhang et al., 2014; Clewell et al., 2014) and PPARα signaling in primary hepatocytes (McMullen et al., 2014).

The report was also instrumental in organizing the Organization of Economical Collaboration and Development (OECD)-level systematic work under the heading of Adverse Outcome Pathways (AOP). The concepts of an AOP framework initially emerged at OECD in the context of ecotoxicology (Ankley et al., 2006), but were soon combined with the Tox-21c concept to extend to all regulatory toxicology. AOP covers exposure to chemical properties, molecular interactions with cells (molecular initiating events), cellular, tissue and organism level effects, and population effects. This representation of our current understanding of toxicological mechanisms, it is important to note, is mainly on a narrative level.

AOP is largely a framework for referencing and assembling existing scientific knowledge into putative pathways. The goal of pathways of toxicity (PoT) (Hartung and McBride, 2011), in contrast, is to develop molecular annotations of network perturbations and their causation from biological high-content phenotyping. Knowledge of these molecular mechanisms is crucial for understanding the chemicobiological interactions on the biological system or the perturbed normal physiology (the homeostasis under stress) which is established in response (Hartung et al., 2012). This is needed to differentiate early molecular initiating events versus homeostatic changes.

## 2 The human toxome vision

Increasingly, technologies enabling broad biological phenotyping of the responses of cells and organisms are emerging that allow elucidating mechanisms of toxicological effects without the necessary prejudice of prior knowledge. These include the various omics and high-content imaging technologies (van Vliet, 2011). These information-rich approaches promise a molecular understanding of toxicological mechanisms and are the starting point of the Human Toxome Project, which aims to develop a process for deducing molecular PoT from toxicological test systems challenged with reference toxicants employing omics technologies. Concepts for annotation and validation of PoT are being developed to establish the “Human Toxome Knowledge-base” and its governance to enable the scientific community to share PoT information.

A number of challenges are discussed below. The concept of PoT itself is a hypothesis, i.e., are there a limited number of pathways which are conserved between cell types, organ systems and even species, as well as characteristics for toxicant classes and hazard manifestations? Challenges include the quality and standardization of the toxicological test systems (especially *in vitro* systems) and omics technologies. The bioinformatics tools for identification, annotation, proof of causality/validation, and link to adversity of PoT are not yet available.

Tackling these questions, a consortium of six partners from Agilent Inc., Brown University, Georgetown University, the Hamner Institute, Johns Hopkins University and the US EPA, funded by a NIH Directors’ Transformative Research Grant (#RO1ES020750), is in its third year of work. The project is unusual in that it is only developing many of the concepts to address these challenges while exploring and further developing, in parallel, the necessary technologies. A number of workshops and commissioned white papers complement the technical work. From the beginning, the project achieved high visibility (Perkel, 2012; Baker, 2013), including a two-hour session in the European Parliament in Brussels (Lunch Debate, May 15, 2012).

## 3 Endocrine disruption as a pilot

Human exposure to environmental estrogenic chemicals (xenoestrogens) is widespread and testing for their effects is a high priority for regulatory agencies. The possible effects include altered development *in utero* through puberty and beyond, as well as effects on reproductive tissues and the development and progression of cancer, especially breast cancer. Environmental chemicals capable of estrogenic endocrine disruption include various organic pollutants such as polychlorinated biphenyls (PCBs), pesticides, dioxins, aromatic hydrocarbons, and various natural chemicals (such as genistein). In particular, there is great public concern about bisphenol A (BPA). Some studies in animal models have shown effects of low dose *in utero* exposure to xenoestrogens (such as BPA) to be associated with abnormal fetal reproductive tract development in male and female offspring (Soto et al., 1995) and mammary tumor development in rats (Acevedo et al., 2013). It remains controversial, however, whether the low dose exposures to xenoestrogens in humans are associated with adverse health effects.

The exact mechanisms through which xenoestrogens affect biological systems are not clear. Estrogenic effects on gene transcription are mediated, it has been thought, by binding to the nuclear estrogen receptors alpha (ERα) and beta (ERβ). More recently, splice variants of ERα66, ERα36 and 46 and ERβ variants ERβ1–5 were identified (Thomas and Gustafsson, 2011). Furthermore, estrogens also have been shown to have rapid effects mediated through a membrane G-protein coupled receptor identified as GPR30 or G-protein-coupled receptor 1 (GPER1) (Thomas et al., 2005). While xenoestrogens bind to the estrogen receptor, binding affinity is typically very low (one-thousandth to one-ten-thousandth that of estradiol), suggesting that – at low levels – these chemicals may not cause adverse outcomes primarily through estrogen receptor mediated mechanisms. Recent studies reveal that several chemicals have a strong binding affinity to another estrogen receptor-related receptor (ERR)γ (Lapensee et al., 2009; Okada et al., 2008).

The estrogenic activities of many compounds have been examined in *in vitro* systems using fluorescent reporters (Bolger et al., 1998; Miller et al., 2000; Busbee et al., 2000; Bovee et al., 2004; Hoogenbooma et al., 2004) and cell proliferation assays (Soto et al., 1995). These assays, however, only provide information on a single endpoint and not the underlying pathways. More recently, microarrays have been used to determine gene expression induced by estrogens (Huan et al., 2014) and metabolomic patterns of metabolite changes (Kolle et al., 2012). However, these endpoints have not been systematically integrated to elucidate classical nuclear and non-classical cytoplasmic/membrane estrogen receptor-mediated (or other) pathways. Thus, it becomes important to develop an approach combining transcriptomic and metabolomic analysis – and later expand to further platform technologies – of the response to estradiol and xenoestrogens to discover PoT using relevant human cell lines such as MCF-7 and T47D (Notas et al., 2012).

Endocrine disruption was chosen as the pilot for the human toxome because of the urgency to complement current risk assessment approaches (Juberg et al., 2014) and the fact that many endocrine system molecular pathways are known. This allows comparison of the PoT (deduced in an untargeted way) with established toxicity pathways.

## 4 Project description

**Fig. 1:**
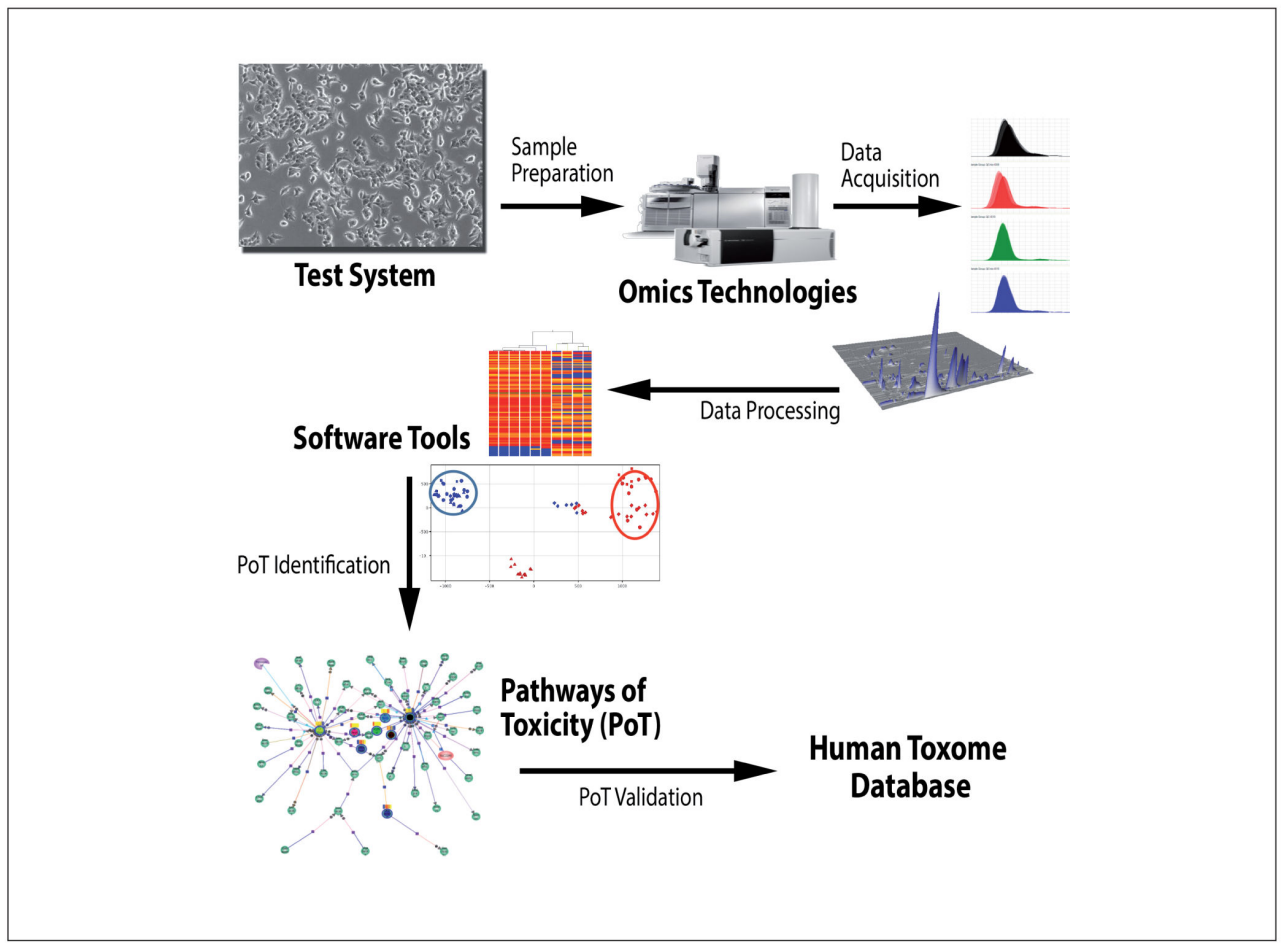
Components of the project “Mapping the Human Toxome by Systems Toxicology” (http://humantoxome.com)

The project “Mapping the Human Toxome by Systems Toxicology” (http://humantoxome.com) aims to map PoT using estrogenic endocrine disruption (ED) as a test case. The central goal is to define, experimentally verify and systematically annotate ED PoT. The overall strategy is to use omics (initially, transcriptomics and metabolomics) to map and annotate PoT, to develop software and visualization tools for integration and analysis of the multi-omics data streams, and to identify, annotate and validate PoT. The project will develop a consensus framework and a community database enabling toxicologists to map the human toxome. The components of the project are illustrated in Figure 1.

The establishment of the quality-controlled cellular test system and definition of toxic treatment was done in the first two years. Two independent labs were responsible for establishing the test model system and providing the biological material. In parallel, and continuing in year 3, the SOP for omics and their performance assessment took place. In order to generate the PoT, transcriptomics and metabolomics experiments are conducted in parallel in two additional labs. Throughout the project, software tools development and data analysis are supported. The definition of the concept of PoT, their identification and validation started in year 2. A series of workshops developing the concepts is a key component of the project – for example, a workshop which developed the following working definition of a PoT (Kleensang et al., 2014):

A Pathway of Toxicity is a molecular definition of the cellular processes shown to mediate adverse outcomes of toxicants.

All data and metadata (i.e., experimental descriptors) are made accessible to the consortium via a centralized cloud server. The last two years of the project include the establishment of the Human Toxome Knowledge-base.

## 5 Project challenges

### Challenge 1: Cell model and reference compound selection

Cell models are prone to artifacts (Hartung, 2007). Very few were successfully validated as reproducible and replicating the responses of animals or humans. In order to increase the likelihood of identifying relevant PoT, work was based on assays that have undergone prevalidation, i.e., reproducibility has been demonstrated and there is evidence for predictive capacity. MCF-7 cells are the test system of the prevalidated protocol for an *in vitro* endocrine disruptor assay (ICCVAM, 2006), and work has thus included mapping PoT in this cell line. Complementary work uses the human breast cancer cell line T47D, which is also part of ToxCast™. Both cell lines express ERα and some ERβ, MCF-7 much lower than T47D. To date, the consortium has focused on the standardization of protocols and assessment of various endpoints, including proliferation, metabolomics, and gene and protein expression. To test estrogen responsiveness, MCF-7 cells have been treated with several concentrations of 17β-estradiol for different durations. MCF-7 cells have demonstrated responsiveness by proliferation and upregulation of known estrogen responsive genes detected by qRT-PCR.

Following exposure to estradiol, the changes seen in these endpoints are reproducible between both laboratories. Following preliminary studies with 17β-estradiol, experiments have focused on the use of receptor-specific selective agonists for both receptors, using propylpyrazole triol (PPT) to target ERα specific pathways and diarylpropionitrile (DPN) to target ERβ. In this system, MCF-7 cells demonstrate high responsiveness to treatment with PPT, with results reproducible between labs, but low to no response to the ERβ agonist DPN. This lack of ERβ activation may be due to the low level of ERβ in the MCF-7 cells. Ongoing work focuses on the ERα pathway and on generating samples for pathway mapping for common estrogenic endocrine disruptors with relevant human exposure, including BPA, genistein, zearalenone and nonylphenol. Additionally, work is moving forward with the T47D cell line, focusing on the ERβ pathway.

### Challenge 2: Cell model standardization and QA

Cell models differ physiologically from their *in vivo* state in many aspects – we take cells out of their natural environments (chemico-physical and biological, such as disruption of cell-cell interactions), which we reproduce poorly in culture. We select the more robust and adaptable cells by providing non-homeostatic conditions favoring growth over differentiation (Hartung, 2007). Furthermore, we do a poor job in standardizing and documenting the experimental conditions. Quality control for cell culture, especially in research and development settings, is in its infancy, for example, with respect to cell authentication, mycoplasma infection, etc. (Hartung, 2013).

The Human Toxome Project makes use of the *Good Cell Culture Practice* (GCCP) guidance (Gstraunthaler and Hartung, 1999; Hartung et al., 2002; Coecke et al., 2005). GCCP acknowledges the inherent variation of *in vitro* test systems and calls for standardization (Leist et al., 2010). In comparison, the *Good Laboratory Practice* (GLP) framework of OECD for regulatory studies gives only limited guidance for *in vitro* work (Cooper-Hannan, 1999), though some parts of GCCP have been adapted into GLP (OECD, 2004).

The quality assurance of the Human Toxome Project further draws on the experience from validation of *in vitro* systems (Hartung et al., 2004; Hartung, 2010; Leist et al., 2012). Especially, the definition of the tests in Standard Operating Protocols (SOP) was continuously updated as part of the Study Plan.

Standardization of *in vitro* systems was a major challenge and continued for over two years: In addition to SOP development, it required exchange of technical personnel and joint training, use of the same stock and passages of cells, and harmonization of all cell culture materials. A first major result was standardization of cell cultures as assessed using two orthogonal global omics technologies, i.e., whole genome transcriptomics on gene arrays and untargeted mass-spectrometry-based analysis. Quite amazingly, cultures in both laboratories were quite reproducible among technical replicates. They were similar within one laboratory between experiments but almost completely different for omics readouts between the laboratories despite all efforts for standardization. This does not reflect – at least not to the same extent – experiences made with either primary or stem cell-based systems. At least in part, it seems to be a problem of the MCF-7 cells, which, after a promising test development for ED screening (ICCVAM, 2006) (the basis for selecting the model for this project) failed the validation study due to reproducibility issues (ICCVAM, 2012) parallel to the running of the Human Toxome Project. In fact, the lack of reproducibility of gene transcription in MCF-7 cells was demonstrated earlier (Ochsner et al., 2009) in a meta-analysis of ten studies all treating MCF7 cells with estradiol: When evaluating the extent of overlap of regulated genes, not a single of the 27,000 transcripts was significantly changed in the same direction in all experiments.

This analysis was expanded by our group where a weighted correlation network analysis indicated that there was substantial similarity in terms of the overall network topography as well as conserved modules of co-regulated genes under estrogen treatment, which suggests that a pathway approach is preferable to analyzing individual differentially expressed genes. This is in line with the results of a consensus report between the EU and US validation bodies, ECVAM and ICCVAM (Corvi et al., 2006), which did not recommend *in vitro* tests using gene array based transcriptomics as an endpoint for validation.

**Fig. 2:**
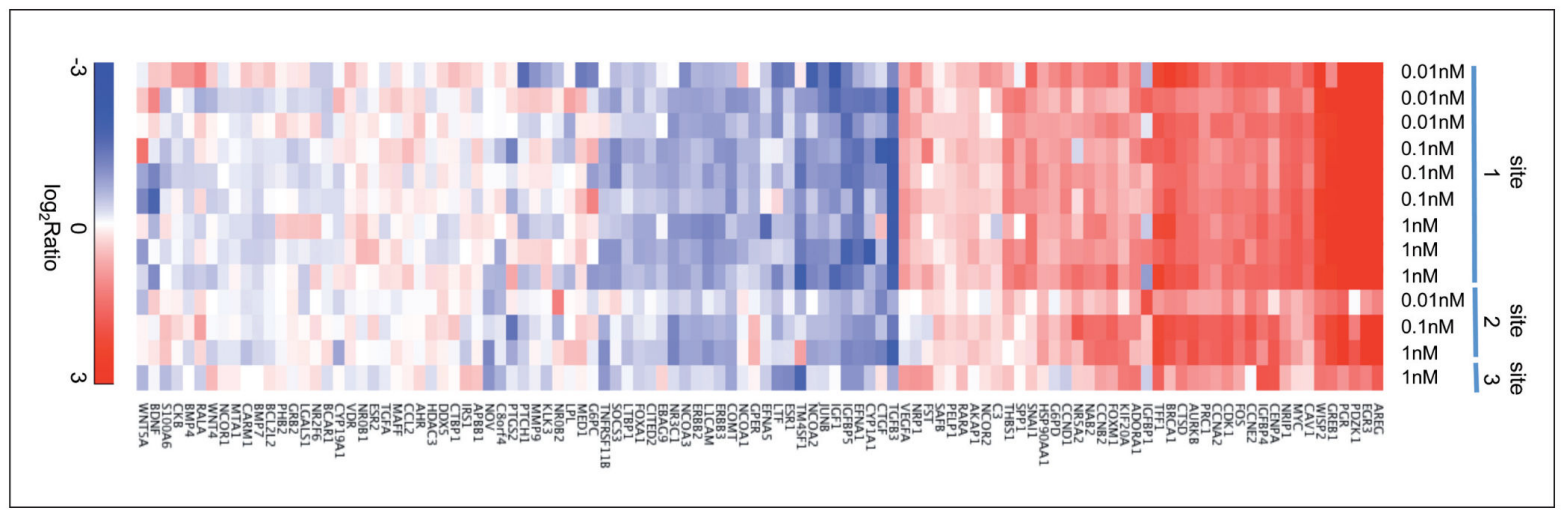
Good correlation between three laboratories in the targeted analysis of 108 genes related to estrogenic effects

Most encouragingly, however, we were able to show that a targeted analysis of 108 genes related to estrogenic effects showed very good correlation between three laboratories (Fig. 2). The overall responses for these genes were similar regardless of site, which means that in principle we can deduce specific responses, but only within very high background noise owing to variability. This is facilitated by gene ontologies, which allow targeting analysis or cluster results on the respective pathways; notably, however, we lack such a metabolite ontology for metabolomics.

### Challenge 3: Omics and quality assurance

Mapping PoT by combined omics technologies requires the integration of data acquired from different platforms (e.g., transcriptomics and metabolomics and, in the future, epigenomics, genomics, proteomics, miRNA omics, etc.). It is important to evaluate the corresponding changes in transcripts and metabolites at different time-points, doses/concentrations, and associated toxicological effects. Metabolomics measures the small molecules of metabolites (which are closest to the phenotype) and therefore has a wide application in toxicity studies (van Vliet et al., 2008; West et al., 2010; Bouhifd et al., 2013). However, despite substantial progress in metabolomics, it still faces a number of challenges. First, it is challenging to generate reproducible data even with the same set of samples when using different approaches for data analysis, which indicates an urgent need for standardization of analytical approaches. There are also continuous challenges with the bioinformatics tools and a serious need for data reduction during metabolomics data processing and tools for metabolite identification. At the current stage, annotation mainly relies on comparing m/z values and retention times with those of known standards run under identical conditions. Although there are several public databases (e.g., METLIN, HMDB) there is still a long way to go towards the development of a useful common database for identification.

The new “omics” era challenges us to put huge amounts of data from different platforms together to interpret them in the context of biology, but the lack of adequate bioinformatics tools is a bottleneck. New open source software and commercial solutions for multiomics data integration, however, are becoming available and facilitate the elucidation of PoT (Connor et al., 2010; Waterman et al., 2010). This systems biology approach is one of the most exciting prospects.

Transcriptomics is one of the major omics approaches for mapping PoT. Transcriptional responses to exposure by environmental xenobiotics can be integrated with other omics approaches to develop an understanding of PoT (Weinstein, 2004). In this stress response network, transcriptional factors are the central mediators for both receiving signals and regulating expression of the downstream genes. Transcriptomic approaches provide mechanistic information by assessing gene expression of known molecular pathways. The strategy involves developing a reference database of transcriptomic profiles with well-characterized pathways using model agents and identifying gene expression patterns associated with the specific pathways and transcriptional factors (Li et al., 2007; Goodsaid et al., 2010). Clusters of genes can be identified that reflect coordinate induction by similar stress agents (Goodsaid et al., 2010), which represent underlying shared signaling pathways.

An important aspect of this project is quality assurance. The relevance and reproducibility of the *in vitro* omics data depends heavily on the quality of the test system and the analytical methods (Ramirez et al., 2013). Fit-for-purpose quality measures for omics-based test methods have been devised (van Vliet et al., 2008). In particular, one of the challenges is the inter-laboratory variation, which has been reported in many transcriptomics studies (Duewer et al., 2009; Beekman et al., 2006; Reynolds, 2005). In order to achieve a stable and reproducible PoT, it is critical to standardize the treatment conditions – such as cell batch, dose and time – which directly affect the output. If an inappropriate dose is selected, there is a risk that the response will be negligible due to underdosing or obscured by apoptotic and other nonspecific responses due to overdosing. Another often-neglected parameter is selecting the time point that reflects specific responses but minimizes secondary effects. If necessary, multiple conditions can be utilized, then common transcriptional responses are screened for PoT. Our study has indicated that robust inter-laboratory reproducibility can be achieved in transcriptomics studies under properly controlled conditions.

### Challenge 4: PoT deduction by bioinformatics

Many aspects of bioinformatics that apply to any high-throughput biology approach are relevant to establishing PoT in a given biological system. However, there are a number of considerations and bioinformatics challenges that are specific to pathway elucidation in the context of toxicity testing. Tox-21c is as much a study of the molecular mechanisms of toxicity as it is the study of dosage. Understanding how a PoT responds to a stimulus, especially at low doses at which most of the environmental exposures are likely to occur, is essential for practical applications of this approach. Thus, developing a PoT requires a more detailed, mechanistic understanding of observed gene, protein and metabolite expression changes through a combination of the curated pathways (such as KEGG, Biocyc, Wiki, Reactome) and *de novo* correlation and regulatory networks, and therefore a more fine-grained understanding of a biological system. From a bioinformatics perspective, this means that instead of stopping at the level of an abstract connectivity map, predictive modeling of the PoT may be required.

Meeting these challenges involves developing novel bioinformatics approaches and applying them to various high-throughput data streams from the literature and from experiments. Transcriptomics has been in use for over two decades and has well-established analysis tools and standards for best practices and documentation. For metabolomics, the analysis is a nascent field. Measuring the abundance of metabolites has technical challenges as well as data analysis bottlenecks due to relatively under-developed computational methods and data infrastructures. In particular, the relatively sparse annotation data available for metabolites compared to genes will require an approach that can infer networks from data (e.g., correlation networks) rather than depend only on curated pathway maps (Khatri et al., 2012), as well as text-mining (Ananiadou et al., 2010) to fill in database gaps. Emerging metabolomics technologies such as LC/MS-MS promise to dramatically improve compound identification after sufficient compound reference data (Smith et al., 2005) and computational search methods become available.

As with all cell systems (and particularly for MCF-7 cells), one challenge is the variability of the system and the reproducibility of high-throughput results. MCF-7 cells show sensitivity of cellular responses to culture conditions, treatments and other factors, compounded by differences in normalization and analysis methods (which sometimes lead to dramatically different outcomes between studies) but, importantly, we found higher correspondences at the pathway level. This does, however, indicate that one key approach is the establishment of an effective dimensionality reduction of the data so that the noise from both the biological and technical variability does not overwhelm the signal. This will ensure that the derived PoT is not the result of over-fitting to one model or one dataset and that it is robust enough to be expanded to all existing data (Klami and Kaski, 2008).

Because of its multidimensional nature, managing and visualizing data produced in a PoT mapping effort is challenging. Interactive visualization tools (Cline et al., 2007) are useful for mapping changes in transcriptional networks, metabolic networks and functional outcomes across experimental factors. Similarly, commercial bioinformatics software such as Agilent’s GeneSpring^2^ offers a platform to enable simultaneous analysis of transcriptomic, metabolomic, proteomic and several sequencing data types as well as mapping high-throughput data onto curated pathways from popular third-party sources. A unique combination of cutting-edge research tools and customized commercial software will allow PoT mapping to interrogate the data much more intuitively and robustly than using flat representations. Lastly, comprehensive PoT mapping requires robust, transparent, and flexible tools to maintain extensive metadata, QC attributes, primary and derived datasets, and the analysis results generated by the project. As a part of the effort to develop commercial software tailored to the needs of large-scale PoT mapping, the Human Toxome Consortium is developing a tailored Laboratory Information Management System based on Agilent’s OpenLAB ELN, which will be tightly integrated with toxome data analysis software via a dedicated software bridge.

### Challenge 5: The Human Toxome Knowledge-base

The implementation of a more mechanistic approach to regulatory toxicology will require scientific data to be delivered faster, more efficiently and accessibly to regulators and advisory bodies (Hartung, 2009b). The road from primary scientific findings to their effective use by regulators, however, is challenging, and this is especially true when it concerns incorporating new technologies into regulatory practice. The high-throughput and high-content methods that are currently generating most data are mainly omics technologies (Kleensang et al., 2014). These technologies produce enormous amounts of data, but do not allow easy interpretation, both because the technologies generate a lot of noise compared to the signal, and the sheer quantity of data makes “seeing the forest for the trees” difficult.

Existing knowledge is scattered in several scientific disciplines and throughout many publications and databases. Although the scientific community has seen a proliferation of pathway-oriented databases such as KEGG, WikiPathways and Reactome as well as several chemical-centered databases such as ACToR, Toxnet or the Comparative Toxicogenomics Database, these databases are poorly harmonized and links between them are rare. As a result, the content is fragmented, appears in multiple formats, and the databases are developed mostly independently of each other. A comprehensive source of information – from adverse outcomes to molecular targets to chemical structure of a toxicant – does not exist, nor are high-throughput data vetted for reliability with, e.g., Klimisch scores (Schneider et al., 2009). The existing databases aggregate as much data as possible with little attention to the quality and reliability necessary for the regulatory context. A Human Toxome Knowledge-base will require more GLP-like documentation, as well as evaluation of the evidence quality in transparent, consistent and objective ways to identify gaps and leave room for professional judgment and weight-of-evidence approaches – something that could be aided by Evidence-Based Toxicology (EBT) (Stephens et al., 2013).

One key consideration is the necessity of making the data accessible for bioinformatics approaches – and that requires making use of ontologies (Hardy et al., 2012a,b) such as ToxML and SBML-compatible pathway representations and machine-readable data that allow to fully take advantage of the data while maintaining ease-of-use and ease-of-interpretability for regulators.

Lastly, it is critical to ensure that any knowledge-base maintains links and integration to other specialized databases (e.g., Gene Ontology, Metlin, or The Human Metabolome Database) in order to leverage data and be useful to the widest possible audience – and this means ensuring data portability between databases. While this problem has been largely solved for transcriptomics data, other omics approaches suffer from a relatively poor infrastructure, e.g., metabolomics (Barupal et al., 2012).

### Challenge 6: Thresholds of adversity

Traditional risk assessments set regulatory limits based on points of departure (PoD) from apical responses seen in animal studies by applying either linear extrapolation to zero-exposure levels or using multiple safety factors. Even then, there remains considerable debate regarding adaptive/reversible effects and adverse responses. One fundamental challenge for a TT21c strategy is to distinguish stress-related adaptive versus adverse responses at a cellular level. Adaptive responses are reversible cellular alterations, while adverse responses result in long-lasting cellular effects or new susceptibilities. The distinction becomes the tipping point for a regulatory action, such as setting an exposure limit for a chemical. At some point in the dose-response, the protective cellular responses at various levels of organization (structural, transcriptomic, metabolic, etc.) will be overwhelmed with failures aligning to produce an “adverse” effect. This failure, commonly known as the “Swiss cheese model of error analysis”, may provide a framework for investigating the cellular dose-response datasets (Boekelheide and Andersen, 2010; Boekelheide and Campion, 2010).

Cell-based tests have the possibility of multiple assay readouts from minimal alterations to adverse outcomes, but there is considerable difficulty in drawing the line between them. In the interim period before risk assessment embraces specific definitions of adversity for cellular responses, case studies will allow comparison of various cell-based responses with related-to-life responses seen in short-term animal studies (referred to as targeted studies in the TT21c report). In the short-term some level of quality assurance of *in vitro* assays against *in vivo* mechanistic studies will be valuable to gain confidence in the value of cell-based approaches (Thomas et al., 2013).

Completion of the processes of distinguishing adaptive from adverse responses will take time and significant amounts of high-quality data from cellular dose-response relationships following toxicant exposures *in vitro*. Bioinformatic and pathway analysis of the large datasets will lead to the identification of cellular responses (both protective and harmful) that are repeatedly observed in progressive cellular injury (Andersen et al., 2011). These adverse cellular responses coupled with *in vitro*-*in vivo* extrapolation and computational systems pathway modeling will convert points-of-departure for adverse cellular responses to exposure limits (Bhattacharya et al., 2011).

### Challenge 7: Validation of PoT

Validation has been developed (Leist et al., 2010) and internationally harmonized (OECD, 2005) only for *in vitro* systems. Governmental validation bodies exist in Europe (ECVAM), US (ICCVAM/NICEATM), Japan (JaCVAM), Korea (KoCVAM), and Brazil (BraCVAM). Some adaptations to *in silico* approaches have taken place (Hartung and Hoffmann, 2009), but no *in silico* approach has been formally validated by a validation body. FDA has also issued guidance for biomarker qualification^3^ (FDA, 2014). Both frameworks are helpful in establishing whether PoT are derived from a meaningful test system and whether a given analyte is predictive for a certain health effect. Neither addresses the aspect of causality or the confirmation of a chain of events leading to adverse outcome, however.

Validating a PoT means establishing the causality between toxicant and hazard manifestation and identification of how this happens. This is difficult on the level of correlation, because we typically do not have the human data for a statistically sufficient number of substances. However, we have growing knowledge of the mechanisms relevant to human health effects. Thus, the efficacy to cover relevant mechanisms for human health and environmental effects is becoming increasingly important. We have called this “mechanistic validation” (Hartung et al., 2013a). Mechanistic validation requires that we establish causality for a given mechanism to create a health or environmental effect. Initially, the classical frameworks of the Koch-Dale (Dale, 1929) and Bradford Hill (Hill, 1965) principles for assessing evidence of causation come to mind. Dale translated the Koch postulates for a pathogen to cause a certain disease to a mediator (at the time histamine as neurotransmitter) of a physiological effect. We can similarly translate to a PoT being responsible for the manifestation of an adverse cellular outcome of a toxicant. Similarly, the Bradford-Hill criteria can be applied. Whether this offers an avenue to systematically establish causality using large datasets from omics and/or high-throughput testing, needs to be explored. It might represent an alternative to the choice of meaningful biomarkers (Blaauboer et al., 2012), which is limited to the current state of knowledge.

The first goal of validation is to demonstrate reproducibility; it should not be a major problem to translate this to PoT. It would require first demonstrating the reproducibility of the results that led to PoT deduction. Arguably, the reproducibility of PoT involvement for a number of toxicants for which the same PoT is claimed needs to be shown. Furthermore, this might require demonstrating that PoT involvement can be shown by orthogonal techniques.

The problem of validation of new approaches such as PoT is the lack of a point of reference to establish the relevance of the approach. Traditionally, an animal experiment has been chosen as point of reference – which also is problematic (Hartung, 2008; Hoffmann et al., 2008). However, there is no animal model for a given PoT; therefore scientific relevance was suggested as a measure to validate new approaches (Hartung, 2010). This mechanistic validation suggests a systematic review of consistency using the scientific literature. Such systematic reviews for toxicology are currently being developed in the context of EBT (Hoffmann and Hartung, 2006), a quality assurance and consensus process modeled on Evidence-based Medicine (EBM) (Hoffmann and Hartung, 2005; Hartung, 2009c).

### Challenge 8: Implementation

We are facing a series of challenges in addition to finding a technical solution, especially for PoT mapping, to put the vision of TT21c into practice (Basketter et al., 2012; Leist et al., 2014). These include the standardization and quality assurance of novel methodologies (Hartung and Daston, 2009), their formal validation, their integration into test strategies (Hartung et al., 2013b) (including threshold setting), and finally global acceptance and implementation (Hartung, 2009b). This will require intense conceptual steering to fit all the pieces of the puzzle together.

A key aspect not yet discussed is the integration of information in test strategies: Tox-21c relies on breaking risk assessment down into many components, here especially a number of PoT for a given hazard and modeling for *in vitro* to *in vivo* extrapolation. These need to be put together again in a way that allows decision-making, ultimately envisioned as Systems Toxicology by simulation (Hartung et al., 2012). Before this, an Integrated Testing Strategy (ITS)-like integration (Hartung et al., 2013b) of data is possible. A probabilistic condensation of evidence into a probability of risk (especially for PoT-based approaches) would be a logical approach.

The key to implementation, however, is regulatory acceptance. We have coined the term “postvalidation” (Bottini et al., 2008). It is increasingly recognized that translation into regulatory guidelines and use is now the bottleneck of the process. Change requires giving up something, not adding to it as “valuable additional information.” In order to accomplish this, we need to demonstrate first the shortcomings of the current approach in a most objective way – again, something that might be accomplished by EBT. Second, we need validation of the new approaches, not only of each PoT on its own, but for the risk assessment tools based on them – again, EBT may help here (Hartung et al., 2013a).

Last but not least, acceptance must occur on an international level. In a global economy with globally acting companies, no change will occur until the last important market has transitioned to a new paradigm (Bottini et al., 2007). For this reason, and because of the magnitude of the task, the Human Toxome Project is aiming for an international collaboration. The envisaged Wiki-process of a public knowledge-base, therefore, needs to demonstrate its usefulness and feasibility as well as adequate governance to appeal to the international scientific and regulatory communities.

## Discussion

Traditional regulatory toxicology is not based on mechanism but on correlation of known results with observations. This type of black box approach has its limitations as many uncertainties come with every new test compound (and are typically addressed by increasing sensitivity (accepting false-positives and not false-negatives) and adding safety (assessment) factors of typically 100–1,000 for possible differences from animals to humans). This approach has arguably protected us despite the introduction of thousands of substances (roughly 1,000 pre-marketing notifications per year in the US). Substances are mainly tested for acute and topical effects, however, and we do not know how well this has protected us against chronic and systemic effects, as only drugs are followed-up for possible health effects when they are on the market.

The explosion of knowledge in life sciences and new technologies with systems level data makes it attractive to use mechanistic approaches. So far, these approaches have been limited to species-specific toxicity – to make the case that certain effects observed in animals are not relevant to humans in order to pursue a product.

The principal question arises: What is a toxicological mechanism? The question is simpler than the answer, because we are looking into highly dynamic networked systems. It is difficult to distinguish where normal response, defense, and adversity start. Most substances are promiscuous in the sense that they have not only one target for interaction with the biological system (molecular initiating event) but they will perturb more and more downstream pathways with increasing dose or duration of exposure. It is difficult to identify which is the pace-making (causal) pathway of a hazard manifestation. Further complications arise from the fact that an organism is a moving target, i.e., developmental processes, adaptive processes, cyclical processes, aging and degeneration all interfere with the perturbation under study. This is especially problematic if we look for the more subtle effects of low-dose chronic exposures. Next, it is not clear how much variance we face: Are these pathways sufficiently conserved between cells, species, or for a given group of toxicants employing the same mechanism? Last but not least, will we still see the PoT at work when dealing with the real-life exposures to mixtures?

All these questions can only be answered by simply doing it. Many iterations and refinements will be necessary. Only when we have a first PoT annotated will we be able to see where and when it works and whether its definition needs to be adapted. Every journey starts with the first step, and these are the first humble steps toward a human toxome. Many more similar and hopefully coordinated projects will be necessary to complete the journey.

## Footnotes

1 http://www.thehamner.org/tt21c, last accessed Sep 22, 2014

2 http://genespring-support.com/, last accessed Sep 22, 2014

3 http://www.fda.gov/Drugs/DevelopmentApprovalProcess/DrugDevelopmentToolsQualificationProgram/ucm284625.htm, last accessed Sep 22, 2014
